# Peripheral Biomarkers of Response to Ketamine and Esketamine Treatment in Unipolar Depressive Disorder—A Scoping Review

**DOI:** 10.3390/jpm16100488

**Published:** 2026-09-23

**Authors:** Aleksandra Metelska, Wojciech Ziemichód, Hanna Karakuła-Juchnowicz

**Affiliations:** 1 I Department of Psychiatry, Psychotherapy and Early Intervention in Lublin, Medical University of Lublin, Głuska 1, 20-439 Lublin, Poland; 2 Doctoral School, Medical University of Lublin, 20-093 Lublin, Poland

**Keywords:** ketamine, depression, biomarkers, predictors

## Abstract

**Objectives**: A significant problem in the treatment of depressive disorders is the growing number of cases of so-called treatment-resistant depression (TRD). There is evidence of ketamine’s effectiveness in treating TRD, with response rates ranging from 40% to 90%. This review aimed to map available studies on peripheral biomarkers of response to ketamine and esketamine in the treatment of depressive disorders, with particular emphasis on biomarker types, study designs, and identification of gaps in current knowledge. **Methods**: A literature review was conducted in accordance with the PRISMA-ScR guidelines for scoping reviews. PubMed and Embase databases were searched from their inception to 1 February 2026. The search terms “ketamine” or “esketamine” were cross-referenced with the terms “depression,” “biomarkers,” and “predictors.” Articles written in languages other than English, unavailable full-text articles, and animal studies were excluded. A manual search of the bibliographies of existing reviews was also conducted to identify additional studies. **Results**: Ultimately, 46 articles met the inclusion criteria for the review: 17 randomized controlled trials, 25 non-randomized interventional studies, 2 observational studies, 1 case study, and 1 case series. Most studies focus on BDNF, inflammatory markers, particularly IL-6, TNF-α, IL-10, and IL-8, and kynurenine pathway metabolites. However, the results of these studies are inconsistent. Other areas remain only fragmentarily explored. **Conclusions**: Significant heterogeneity was observed in study designs, analytical methods, and definitions of clinical response. No peripheral biomarker has yet demonstrated sufficient consistency for clinical implementation. Future studies should prioritize standardized, adequately powered prospective designs and multimarker approaches integrating neuroplastic, inflammatory, and metabolic pathways, with particular attention to esketamine-treated populations.

## 1. Introduction

According to the most recent World Health Organization estimates, approximately 322 million people worldwide experienced depression in 2023, corresponding to 4% of the global population and 5.2% of adults [1]. Another major problem is the growing number of cases of treatment-resistant depression (TRD). Although there is currently no universally accepted definition, it is typically defined as the lack of symptomatic remission after treatment with at least two antidepressants at an appropriate dose for an appropriate period of time [2]. Epidemiological estimates vary depending on the definition of treatment resistance and the population studied; nevertheless, TRD is generally estimated to affect approximately 30% of patients with MDD [3].

Increasing levels of treatment resistance are associated with higher direct and indirect costs and worse overall health status [4]. Compared with other patients with MDD, individuals with TRD experience poorer quality of life, greater impairment in daily activities and work productivity, and higher healthcare resource utilization [5]. A large population-wide cohort study demonstrated that, compared with non-TRD episodes, TRD was associated with approximately three times as many inpatient bed-days, more than twice as many lost workdays, a more than four-fold higher occurrence of intentional self-harm, and a 23% higher risk of all-cause mortality [6].

The literature provides evidence for the effectiveness of ketamine and esketamine in the treatment of major depressive disorder (MDD). Ketamine has been used in numerous clinical trials in patients with TRD, with response rates ranging from 40% to 90% [7]. Intravenous infusion of ketamine produced a rapid antidepressant effect within a few hours of administration and lasted from 2 to 14 days [8].

Ketamine is a noncompetitive antagonist of the N-methyl-D-aspartate (NMDA) receptor. Its antidepressant effects are mediated by its effects on the glutamate system. Glutamate is an excitatory neurotransmitter that influences neurodevelopment and neuroplasticity and participates in neurocognitive functions. Dysregulation of neuroplasticity may contribute to the development of psychiatric disorders, including depressive disorders [9]. Esketamine, the S-enantiomer of racemic ketamine, was approved by the FDA in 2019 for the treatment of treatment-resistant depression [10].

The literature on the subject presents several potential mechanisms of action for ketamine, which likely operate in a complementary manner. Ketamine binds to the NMDA receptor (NMDAR) on presynaptic or postsynaptic glutamatergic neurons, as well as on GABAergic interneurons, thereby blocking and inhibiting NMDARs. This triggers a cascade of events: decreased γ-aminobutyric acid concentration, glutamate release, activation of α-amino-3-hydroxy-5-methylisoxazole-4-propionic acid receptors, release of brain-derived neurotrophic factor (BDNF), and activation of tropomyosin receptor kinase B and the mammalian target of rapamycin complex 1 (mTORC1) [9]. As a result, signaling pathways associated with neuroplasticity are activated, resulting in long-term synaptic potentiation and stimulation of synaptogenesis. These mechanisms are considered the basis of ketamine’s efficacy [11,12]. The neurobiological basis of non-response to ketamine remains poorly understood. Differences in the engagement of glutamate-dependent neuroplasticity and in the modulation of neurotrophic, inflammatory, and kynurenine pathways have been proposed; however, these hypotheses are based mainly on preclinical and exploratory biomarker studies, and none have been conclusively confirmed [13,14]. Prospective and retrospective studies indicate that a shorter duration of untreated illness is a predictor of better treatment response and better long-term outcomes. These data apply to both the first episode and subsequent episodes [15,16,17]. Nevertheless, reliable predictors of response to ketamine and esketamine have not yet been established, highlighting the need to identify biomarkers that could facilitate treatment selection and personalization [18].

This review aimed to map available studies on peripheral biomarkers of response to ketamine and esketamine in the treatment of depressive disorders, with particular emphasis on biomarker types, study designs, and identification of gaps in current knowledge. The analysis was limited to peripheral biomarkers defined as measurable biological indicators determined in biological material collected peripherally. The review includes, among others, neurotrophic, inflammatory, metabolic, and neuroendocrine markers, kynurenine pathway metabolites, and indicators related to intracellular signaling pathways assessed in the context of response to ketamine or esketamine treatment.

The following research questions were addressed:

1. Identification of previously studied peripheral biomarkers in the context of response to ketamine and esketamine.
2. What types of research designs dominate the literature?
3. What research gaps are identified in the current state of knowledge?

## 2. Methods

### 2.1. Review Design and Search Strategy

A scoping review was conducted according to the PRISMA-ScR guidelines for scoping reviews [19]. The aim was to characterize existing studies on biomarkers of response to ketamine and esketamine. The PubMed and Embase databases were searched from their inception to 1 February 2026. The search strategy was based exclusively on free-text terms, and no database-specific controlled vocabulary terms, such as MeSH headings in PubMed or Emtree terms in Embase, were explicitly incorporated. The same Boolean search string was applied in both databases: (ketamine OR esketamine) AND depression AND (biomarker* OR “biological marker*” OR predictor*).

All retrieved articles were screened for inclusion in this review based on their title and abstract. Search results that were irrelevant to the scope of this manuscript were excluded from further analysis. Following the electronic database search, reference lists of relevant publications were manually screened to identify additional eligible studies. Bibliographies were also hand-searched to identify additional relevant studies.

The search strategy, adapted to the purposes of this review, enabled the inclusion of a wide range of studies. Clinical trials with various designs (RCTs, non-randomized interventional studies, observational studies, open-label studies, case reports, and case series) were included, without formal quality assessment of the evidence. To provide the broadest possible representation of existing reports on biomarkers of response to ketamine and esketamine, conference abstracts presenting original research results were also included in this review; these are marked accordingly in the table (Table 1). The protocol for this scoping review was not prospectively registered or published.

### 2.2. Inclusion and Exclusion Criteria

The following inclusion criteria were adopted for the review:

(1) studies written in English;
(2) participants with a current depressive episode in the course of major depressive disorder (MDD), including treatment-resistant depression, diagnosed according to the criteria applied in each study;
(3) esketamine or ketamine therapy for the reported depressive episode;
(4) studies that assessed biomarkers measured in biological material collected peripherally in the context of ketamine or esketamine treatment.

The following exclusion criteria were applied:

(1) depressive episode in the course of bipolar disorder or mixed populations without a clear division into MDD/BD groups;
(2) studies focusing solely on clinical, cognitive, neuroimaging, or neurophysiological markers, without assessing peripheral biomarkers;
(3) studies that could not distinguish the effect of ketamine or esketamine from other therapeutic or neuromodulatory interventions;
(4) animal studies;
(5) studies that assessed subjects in a surgical/perioperative setting.

### 2.3. Study Selection Process

The PubMed and Embase databases were searched from their inception to 1 February 2026. Records from both databases were exported in Citation Manager (NBIB) format and imported into the Rayyan platform [20], where duplicate records were identified and removed before the study selection process began. Potential duplicates were automatically identified by the program and then verified by the researcher before being removed. Verification was based on bibliographic data matching, including titles, findings, publication editions, and DOI numbers.

The search yielded 441 records in PubMed and 989 records in Embase. A total of 1430 records were identified, including 365 exclusions as duplicates. Based on the title and abstract, 935 records were deemed irrelevant or met explicit exclusion criteria. In total, 130 articles were eligible for full-text review, of which 2 were excluded due to: unavailability of the full text (*n* = 1) and the full text being available in a language other than English (*n* = 1). The remaining 128 records were submitted for full-text review, 35 of which met the inclusion criteria for review. To expand the scope of identified sources and minimize the risk of omitting detailed research, manual searches of the reference lists of the available articles were conducted. This procedure was followed, and 11 additional publications were included (Figure 1).

A total of 46 articles ultimately met the inclusion criteria and were included in the review. Only original empirical studies (randomized controlled trials, non-randomized interventional studies, observational studies, and conference abstracts) were included in the analysis. Conference abstracts are included only if the inclusion criteria were met and included data regarding the study specification, the use of ketamine or esketamine, and recommended biomarkers. Conference abstracts were excluded if the available information was insufficient to assess eligibility according to the inclusion criteria or to extract data relevant to the objectives of the review. Review articles, meta-analyses, editorials, commentaries, and study protocols without reported results were also excluded. The study selection process was conducted independently by two reviewers. The reviewers were not formally blinded to each other’s decisions. Any disagreements were resolved through discussion until consensus was reached.

In this review, we focused exclusively on synthesizing the available evidence on potential biomarkers of treatment response to ketamine/esketamine in unipolar depressive disorders, including treatment-resistant depression. The use of the PRISMA-ScR guidelines enhanced the transparency and methodological rigor of the review, enabling a systematic mapping of the available evidence and the identification of existing research gaps in the field.

### 2.4. Data Charting and Synthesis

Data from the included studies were charted by one reviewer in a structured format based on the objectives of the review. One reviewer extracted the data, and a second reviewer verified the extracted information against the original publications. Any discrepancies or uncertainties were resolved through discussion and consensus. The charted variables included the first author and year of publication, study title, study design, sample size, diagnostic group, ketamine or esketamine treatment regimen, investigated biomarker, biological material, biomarker assessment time points, definition of clinical response, and main findings relevant to treatment response. For conference abstracts, only information explicitly reported in the available abstract was extracted, and missing methodological details were not inferred. The included studies were grouped according to the biological pathway or biomarker category investigated. Because of substantial heterogeneity in study designs, treatment protocols, biomarker assessment methods, sampling time points, and definitions of clinical response, the findings were synthesized descriptively, and no quantitative meta-analysis was performed.

During the preparation of this manuscript, the authors used AI-assisted tools to support language editing, improve the clarity and readability of the text, refine the wording and structure of selected passages, and assist in the preparation of tables based exclusively on data extracted and verified by the authors. The AI tools were not used for literature searching, study selection, data extraction, data analysis, or the generation of scientific findings or conclusions. All AI-assisted content, including the tables, was critically reviewed, verified, and edited by the authors, who take full responsibility for the content of this publication.

## 3. Results

### 3.1. Characteristics of the Included Studies

Following the full-text analysis of the selected articles, a total of 46 articles were ultimately included in the review: 17 randomized controlled trials (including 5 conference abstracts presenting original results), 25 non-randomized interventional studies (including 8 conference abstracts presenting original results), 2 observational studies, 1 case study, and 1 case series. A summary of the results is presented in Table 1.

Among the studies included in the review, considerable heterogeneity was observed with regard to the analytical methods used for biomarker assessment, the biological material analyzed, the ketamine or esketamine administration regimen, the time points used to assess treatment response, and the biomarkers analyzed. Biomarkers were investigated for their potential utility as predictive markers (predicting the response to ketamine or esketamine treatment) and dynamic markers (changes in biomarker levels following the therapeutic intervention).

The available literature was dominated by studies focusing on biomarkers related to neuroplasticity and inflammation, whereas metabolomic markers, proteomic markers, and individual signaling proteins were investigated only sporadically. The following biomarkers were investigated in the included studies: BDNF (*n* = 15), inflammatory parameters (*n* = 12), kynurenine pathway metabolites (*n* = 7), neuroendocrine markers (*n* = 6), mTOR (*n* = 4), GSK-3 (*n* = 2), metabolomic markers (*n* = 2), D-serine (*n* = 1), RANKL (*n* = 1), VEGF (*n* = 1), FGF-2 (*n* = 1), S100B (*n* = 1), arginine (*n* = 1), sphingomyelins (*n* = 1), P11 (*n* = 1), proteomic markers (*n* = 1), and opioid receptors (*n* = 1). Some of the included articles evaluated the association between more than one biomarker and the therapeutic response to ketamine or esketamine within a single publication. To facilitate comparison of findings across studies, the current evidence was grouped according to peripheral biomarker categories and is summarized in Table 2. A detailed summary of the peripheral biomarkers investigated across the included studies is provided in Supplementary Table S1.

### 3.2. Categories of Peripheral Biomarkers Investigated

#### 3.2.1. Neurotrophic and Growth Factors

Neurotrophic factors, particularly BDNF, were the most frequently investigated biomarkers in the analyzed publications. Individual studies also evaluated growth factors with neurotrophic properties, such as VEGF (*n* = 1), FGF-2 (*n* = 1), and S100B (*n* = 1). All of these studies examined the association between these biomarkers and the response to intravenous ketamine infusion.

BDNF was evaluated in 15 of the 46 included studies. Eight studies were non-randomized interventional studies, including two conference abstracts presenting original results. Six of the identified publications were randomized controlled trials, including two conference abstracts, and one publication was an observational study [21,22,23,24,25,26,27,28,29,30,31,32,33,34].

The individual studies differed with respect to both the biological material analyzed (serum vs. plasma) and the timing of biological sample collection for biomarker assessment. The analytical methods included conventional ELISA assays for the measurement of individual biomarkers and multiplex immunoassays.

The analyzed studies assessed baseline BDNF levels as a potential predictive marker or measured changes in BDNF levels following ketamine administration as a potential dynamic marker. The findings regarding changes in BDNF levels after ketamine administration were heterogeneous; some publications reported an increase in BDNF levels at specific time points, whereas others did not demonstrate significant changes. In some of the analyzed publications (*n* = 6), a potential association between BDNF and the therapeutic response to ketamine was demonstrated; however, the findings remain heterogeneous.

Other growth factors, such as VEGF, FGF-2, and S100B, were analyzed only in individual studies. One study investigating FGF-2 demonstrated its potential predictive value in the context of the response to ketamine; however, due to the limited number of publications, it is not possible to identify consistent patterns of association or compare findings across studies [21,25,35].

The identified gaps in the available literature include small sample sizes, lack of standardization regarding the timing of biomarker assessment, and the limited number of randomized studies evaluating BDNF and other growth factors as potential markers of treatment response.

#### 3.2.2. Inflammatory and Immunological Markers

The literature includes studies investigating the following inflammatory markers in relation to ketamine or esketamine treatment: IL-1α, IL-1β, IL-2, IL-5, IL-6, IL-7, IL-8, IL-10, IL-13, TNF-α, IFN-γ, CRP, IL-12p70, IL-17A, IL-17F, IL-23, IL-21, IL-25, sCD40L, MIP-1β, MIP-3α, ITAC, GM-CSF, and fractalkine. A total of 12 of the 46 identified publications investigated the association between inflammatory parameters and the response to ketamine (*n* = 9) or esketamine (*n* = 3). Most of these were non-randomized interventional studies (*n* = 9), whereas only a few were randomized studies (*n* = 3), including one conference abstract. Among the identified publications on inflammatory parameters, the largest number of studies investigated IL-6 (*n* = 10), TNF-α (*n* = 9), IL-10 (*n* = 5), and IL-8 (*n* = 4). Additionally, one randomized study investigated the bone turnover marker RANKL [25,30,40,42,48,49,50,51,52,53,54,55,56].

The individual articles differed in terms of study methodology, drug administration regimen, and the timing of inflammatory parameter assessment. Some publications analyzed baseline cytokine levels as potential predictive markers, whereas others focused on dynamic changes following ketamine administration. Some studies indicate an association between parameters such as IL-6, IL-8, IL-1β, IL-10, and ITAC and the therapeutic response to ketamine or esketamine; however, the existing gaps in knowledge (methodological heterogeneity, lack of reproducibility across subsequent studies, and the limited number of randomized controlled trials) do not allow for the identification of consistent patterns of association.

#### 3.2.3. Kynurenine Pathway Metabolites

In 7 of the 46 studies, the potential role of kynurenine pathway metabolites as biomarkers of response to ketamine was evaluated: 4 non-randomized interventional studies (including 3 conference posters presenting original results), 2 randomized controlled trials (including 1 conference poster), and 1 observational study [36,37,38,39,40,41,42].

In the aforementioned publications, the authors assessed baseline levels of kynurenine pathway metabolites as well as their dynamic changes following intravenous ketamine (*n* = 6) or intranasal esketamine (*n* = 1) administration. The most frequently analyzed kynurenine pathway metabolites were tryptophan (TRP), kynurenine (KYN), kynurenic acid (KYNA), quinolinic acid (QUIN), and 3-hydroxykynurenine (3-HK). Some publications also evaluated derived indices, such as the KYN/TRP and KYNA/QUIN ratios. Some articles indicated the predictive value of KYNA with respect to the response to ketamine and esketamine. However, the findings of the individual studies remain heterogeneous, with a lack of consistency in the analytical methods used, the time points of KYNA metabolite assessment, and the drug administration regimens.

#### 3.2.4. Neuroendocrine and Metabolic Markers

A total of six publications on neuroendocrine biomarkers were identified in the literature. All of them evaluated the association between endocrine markers and the therapeutic response to intravenous ketamine infusions [51,53,57,58,59,60].

In three articles, the authors investigated cortisol as a biomarker of response to ketamine (*n* = 3); all of these studies were conducted as randomized controlled trials (two of the publications were presented as conference abstracts). Two studies (one randomized controlled trial and one non-randomized interventional study) evaluated thyroid hormones (fT3 or TSH). In addition, one non-randomized conference abstract investigated modulation of the insulin signaling pathway.

#### 3.2.5. Intracellular Signaling Markers

Individual studies conducted in small study populations evaluated the association between the therapeutic response to intravenous ketamine infusions and intracellular signaling markers, such as mTOR, GSK-3, and p11.

Only four publications investigating the role of mTOR in the response to ketamine were identified in the available literature: one case study, one case series (based on three cases), and two randomized controlled trials (including one conference poster), all conducted in small study populations (10 patients with treatment-resistant depression). The same two randomized studies also evaluated the potential association between ketamine and GSK-3 in the same study populations. Only one randomized study investigating the p11 marker was identified [43,44,45,46,47].

The identified evidence base regarding intracellular signaling markers is limited with respect to the number of studies, study population size, and the diversity of study designs. This indicates the need for further, better-designed studies involving larger study populations.

#### 3.2.6. Other Biomarkers

In addition to the main biomarker categories, the literature included individual studies investigating the following factors: arginine [41], D-serine [61], sphingomyelins [41], opioid receptors [65], metabolomic markers [63,64], and proteomic markers [62]. The existing evidence base regarding these biomarkers is limited and exploratory in nature. Due to the limited number of publications, it is not possible to identify consistent patterns of association or compare findings across studies.

## 4. Discussion

Treatment-resistant depression (TRD) is currently a significant challenge faced by many clinicians. Ketamine and esketamine represent important therapeutic options for patients diagnosed with major depressive disorder (MDD) who do not respond to other forms of treatment. Given the high proportion of treatment-resistant patients, as well as the substantial costs associated with ineffective depression treatment, there is a need to identify biomarkers that could predict treatment response to ketamine/esketamine.

Biomarkers may be considered in two main contexts: as predictive biomarkers, whose baseline values may enable the identification of patients with a greater likelihood of responding to treatment, and as dynamic biomarkers, whose changes during treatment may reflect biological processes associated with the mechanism of action of the drug or the clinical response. In this article, we reviewed the literature on potential biomarkers of treatment response to ketamine/esketamine in depression.

Analysis of the available publications indicates that the potential biological mechanisms underlying the response to ketamine have been explored extensively, although unsystematically. The most frequently investigated biomarkers were BDNF (*n* = 15), inflammatory markers (*n* = 12), and kynurenine pathway metabolites (*n* = 7). Of the 15 studies investigating BDNF, 6 demonstrated its potential as a dynamic biomarker of response to ketamine, while 3 also demonstrated a significant association between baseline BDNF levels and subsequent treatment response.

Among the 12 studies investigating inflammatory markers, 7 observed changes in their levels following ketamine or esketamine treatment. The most frequently analyzed inflammatory markers were IL-6, TNF-α, IL-10, and IL-8. Three studies suggested the potential of IL-6 as a dynamic biomarker, two of which also suggested its potential as a predictive biomarker. It should be emphasized that the remaining studies did not observe significant associations involving inflammatory markers or reported findings in the opposite direction. Studies investigating the kynurenine pathway were less numerous; however, five of the seven studies demonstrated significant changes in TRP, KYN, and KYNA levels following ketamine or esketamine treatment. Numerous other biomarkers were investigated only occasionally, and the available findings originated from a limited number of studies conducted in small study populations and were primarily exploratory in nature.

The available evidence indicates that, among the biomarkers analyzed, the greatest amount of evidence has been accumulated for BDNF, inflammatory markers, and kynurenine pathway metabolites. The relatively frequent observation of changes in these parameters in association with treatment may indicate the importance of neuroplastic, immunological, and metabolic processes in the mechanism of action of ketamine. At the same time, the lack of reproducible findings for individual biomarkers and the limited number of studies demonstrating their predictive value suggest that identifying complex biomarker profiles may currently be of greater importance than searching for a single marker of treatment response. Therefore, a potentially important direction for future research may be the integration of biomarkers representing different biological pathways and their evaluation in combination with the clinical characteristics of patients.

A review of the literature on biomarkers of response to ketamine/esketamine treatment identified several limitations and gaps in the currently available evidence. First, considerable heterogeneity was observed in the methodology of the included studies. The studies differed with respect to the analytical methods used, the biological material collected for biomarker assessment, the drug administration regimen, the time points of biomarker measurement, and the definitions and methods used to assess clinical outcomes. Such substantial methodological differences may generate inconsistent and sometimes contradictory findings, making comparisons and data synthesis difficult and limiting the ability to conclude the clinical utility of individual biomarkers. The included studies also differed in their study designs, often included small study populations, and did not include control groups.

It is also worth noting that the dynamics of peripheral blood biomarkers are influenced by non-psychiatric medical conditions, particularly in the case of inflammatory markers, which further complicates the interpretation of the findings.

Furthermore, studies on intravenous ketamine clearly predominate, whereas data regarding biomarkers of response to intranasal esketamine remain very limited. Only a few publications included patients treated with esketamine (*n* = 5): three studies investigated inflammatory markers, one investigated BDNF, and one investigated the kynurenine pathway. At the present stage, none of the analyzed biomarkers have been validated as a tool to support clinical decision-making.

In accordance with the scoping review methodology, the present review aimed to comprehensively map the available scientific evidence; therefore, conference abstracts presenting original study results were also included in the analysis. Nearly one-third of the included records consisted of conference abstracts (13/46), whose level of evidence is lower than that of full-text publications. Their inclusion made it possible to consider the most recent findings that have not yet been published in full and to reduce the risk of overlooking important research directions. However, it should be emphasized that findings derived from conference abstracts were interpreted with caution because of the limited methodological information available and the absence of a full publication process.

The conduct of the present review was also associated with several limitations. Only publications written in English were included, and the lack of access to the full text of two articles required the authors to exclude potentially relevant studies (*n* = 2). In addition, the review was conducted without the use of automated search methods, which may have introduced a risk of inadvertently overlooking relevant studies. The study selection process was performed using the Rayyan tool, which enables the organization and manual labeling of publications during the screening stage. The literature search was limited to PubMed and Embase and did not include additional databases. Therefore, some potentially relevant studies may not have been identified. Furthermore, the search strategy relied on free-text terms without the use of database-specific controlled vocabulary (MeSH or Emtree), which may have reduced search sensitivity and resulted in some relevant studies being missed.

Another limitation is that the protocol for this scoping review was not prospectively registered.

As a scoping review, the present study did not include a formal assessment of methodological quality or risk of bias of the individual publications. Consequently, the findings of this review do not allow for a formal comparison of the reliability of individual reports or for an assessment of the impact of methodological quality on the observed differences and inconsistencies across studies.

## 5. Clinical Implications

Despite the growing number of studies investigating biomarkers of response to ketamine and esketamine treatment, none have yet been sufficiently validated for use in routine clinical practice. Data regarding biomarkers of response to intranasal esketamine remain particularly limited. Consequently, decisions regarding treatment qualification should continue to be based on clinical assessment rather than biomarker measurements.

Analysis of the available literature indicates the potential importance of BDNF, inflammatory markers, and kynurenine pathway metabolites. At present, their greatest value lies in improving our understanding of the mechanisms of action of ketamine and esketamine and in guiding future research.

Future studies should include larger patient populations, use standardized protocols for biological sample collection and analysis, and apply clearly defined clinical outcomes and assessment time points. A review of the available studies suggests that the search for a single biomarker of response to ketamine or esketamine treatment is unlikely to yield the expected results. A more promising direction appears to be the development of multibiomarker models integrating inflammatory, neuroplasticity-related, and metabolomic markers with detailed clinical characterization of patients. Such an integrated approach may substantially improve the accuracy of predicting treatment response and contribute to the implementation of the principles of precision psychiatry.

## 6. Conclusions

This scoping review presents the current state of knowledge regarding peripheral biomarkers of response to ketamine and esketamine treatment in patients with unipolar depressive disorders. Most studies have focused on biomarkers of neuroplasticity, inflammatory processes, and kynurenine pathway metabolites; however, the available findings remain inconclusive and do not support the identification of a biomarker with established clinical utility. At the same time, many potentially important areas, such as metabolomic markers, proteomic markers, and biomarkers related to intracellular signaling pathways, remain insufficiently explored. The review demonstrated substantial heterogeneity among the available studies, resulting from differences in the characteristics of the study populations, treatment protocols, biological sample collection time points, analytical methods, and definitions of clinical response. This limits the comparability of the findings and hampers the identification of reliable predictive biomarkers.

A review of the available studies suggests that the search for a single biomarker of response to ketamine or esketamine treatment is unlikely to yield the expected results. A more promising direction appears to be the development of multibiomarker models integrating biomarkers of neuroplasticity, inflammatory processes, and metabolomic markers with comprehensive clinical characterization of patients. Such an integrated approach may improve the accuracy of predicting treatment response and contribute to the advancement of precision psychiatry.

The full validation of biomarkers of response to ketamine and esketamine will require well-designed prospective studies involving large patient populations, using standardized therapeutic and analytical protocols as well as consistent criteria for the assessment of clinical response. Increasing the number of studies investigating intranasal esketamine is of particular importance, as biomarkers of response to this treatment remain considerably less well understood than those associated with ketamine.

## Figures and Tables

**Figure 1:**
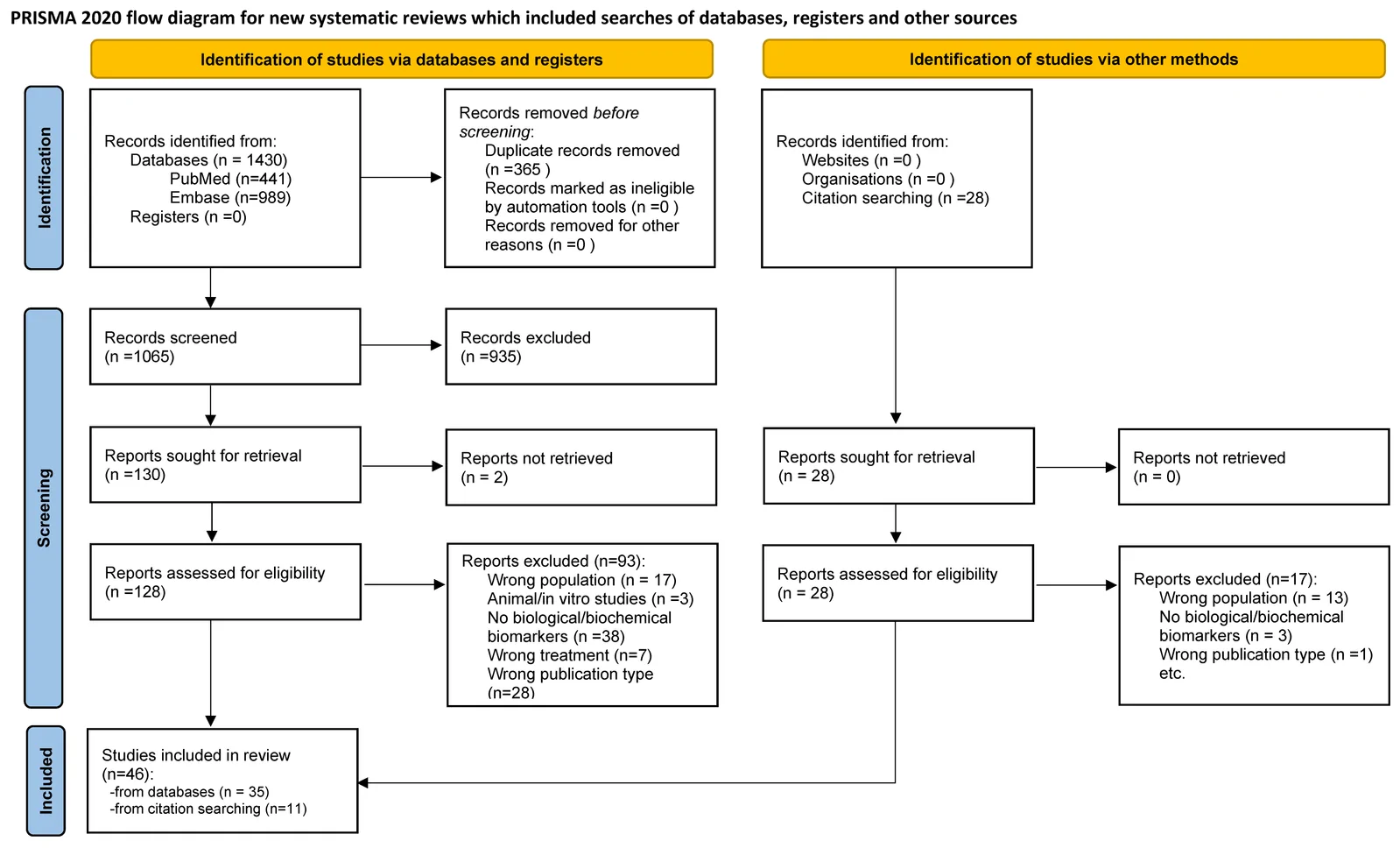
PRISMA 2020 flow diagram of the study selection process, adapted from Page et al, Source: Page MJ, et al. BMJ 2021;372:n71. doi: 10.1136/bmj.n71.

**Table 1:** Characteristics and main findings of studies investigating peripheral biomarkers of response to ketamine and esketamine in major depressive disorder.

Authors	Title	Biomarker	Type of Study	Sample Size	Type of Depression (MDD/TRD)	Intervention	Biological Material	Assessment Time Point	Definition of Clinical Response	Main Results
Jiang H et al., 2021 [21]	Plasma Levels of Brain-Derived Neurotrophic Factor and S100B in Relation to Antidepressant Response to Ketamine.	BDNF, S100B	RCT	30 (20 KET, 10 placebo)	MDD	KET (single infusion)	Plasma	Baseline; 1–2 days post-infusion	≥50% reduction in MADRS score from baseline 24 h after a single KET infusion.	No changes in BDNF or S100B levels; no differences between responders and non-responders.
Machado-Vieira R et al., 2009 [22]	Brain-derived neurotrophic factor and initial antidepressant response to an N-methyl-D-aspartate antagonist.	BDNF	Non-randomized interventional study	23	MDD	KET (single infusion)	Plasma	baseline; 40, 80, 120, 230 min post-infusion	No formal definition of clinical response; changes in MADRS score were analyzed.	No changes in BDNF levels.
Duncan WC et al., 2013 [23]	Concomitant BDNF and sleep slow wave changes indicate ketamine-induced plasticity in major depressive disorder.	BDNF	Non-randomized interventional study	30	TRD	KET (single infusion)	Plasma	Baseline, 230 min post-infusion	≥50% reduction in MADRS score from baseline 24 h after a single KET infusion.	Increased BDNF levels after KET infusion.
Haile CN et al., 2014 [24]	Plasma brain derived neurotrophic factor (BDNF) and response to ketamine in treatment-resistant depression	BDNF	RCT	22 (15 KET, 7 midazolam)	TRD	KET (single infusion) vs. midazolam	Plasma	Baseline, 240 min post-infusion	≥50% reduction in MADRS score from baseline 7 days after the KET infusion.	Greater increase in BDNF levels after KET infusion in responders; negatively correlated with depression severity.
Kiraly DD et al., 2017 [25]	Altered peripheral immune profiles in treatment-resistant depression: response to ketamine and prediction of treatment outcome.	BDNF, FGF-2, IL-1a, IL-b, TNF-a, IL-13, IP-10, IL-8, IL-7	Non-randomized interventional study	33 + 26 HCj	TRD	KET (single infusion)	Serum	baseline; 4 h; 24 h post-infusion	≥50% reduction in MADRS score from baseline 24 h after a single KET infusion.	Lower baseline FGF-2 levels predicted response; no changes in the other biomarkers.
Caliman-Fontes AT et al., 2023 [26]	Brain-derived neurotrophic factor serum levels following ketamine and esketamine intervention for treatment-resistant depression: secondary analysis from a randomized trial.	BDNF	RCT	53	TRD	KET (single infusion) vs. ESK (1x)	Serum	baseline; 24 h; 7 days post-infusion	≥50% reduction in total MADRS score from baseline at any post-infusion assessment.	No changes in BDNF levels; no differences between KET and ESK; no association between BDNF levels and treatment response.
Kang MJY, Vazquez GH et al., 2022 [27]	Association between peripheral biomarkers and clinical response to IV ketamine for unipolar treatment-resistant depression: An open label study.	BDNF	Observational study	15	TRD	KET (10 infusions)	Plasma	Baseline and post-infusions	≥50% reduction in MADRS score from baseline after completion of 10 KET infusions (4 weeks of treatment).	Higher BDNF levels in responders vs. non-responders; higher baseline BDNF levels predicted clinical improvement.
Zheng W et al., 2020 [28]	Association between plasma levels of BDNF and the antisuicidal effects of repeated ketamine infusions in depression with suicidal ideation.	BDNF	Non-randomized interventional study	57	MDD	KET (6 infusions)	Plasma	Baseline, 1, 13, 26 days post-infusion	≥50% reduction in Beck Scale for Suicide Ideation (SSI-Part 1) score from baseline on Day 13 of treatment (1 day after the sixth KET infusion).	No changes in BDNF levels over time; no differences between responders and non-responders; no correlation between BDNF levels and MADRS reduction.
Medeiros GC et al., 2021 [29]	Treatment of depression with ketamine does not change plasma levels of brain-derived neurotrophic factor or vascular endothelial growth factor.	BDNF	RCT	39	MDD	KET (single infusion)	Plasma	Baseline; 230 min; 1, 3 days post-infusion	≥50% reduction in MADRS score from baseline at 24 h.	No significant changes.
Kang MJY et al., 2021 [30]	A Pilot Study: An Open-Label Biomarker Development of Ketamine for Unipolar Refractory Depression	BDNF, IL-6, IL-10, TNF-α	Non-randomized interventional study	15	MDD	KET (repeated infusions	Peripheral blood (unspecified)	3 points (unspecified)	≥50% reduction in MADRS score from baseline at 24 h.	Higher baseline BDNF levels predicted KET response; BDNF levels during treatment correlated with MADRS reduction; no significant changes in the other biomarkers.
Allen AP et al., 2015 [31]	Serum BDNF as a peripheral biomarker of treatment-resistant depression and the rapid antidepressant response: A comparison of ketamine and ECT.	BDNF	Non-randomized interventional study	18 KET vs. 17 ECT, 20 HC	MDD	KET (single infusion) vs. ECT	Serum	Baseline, 1 day post-infusion	No formal definition of clinical response; changes in MADRS score were analyzed.	BDNF levels were significantly higher in responders.
Grunebaum et al., 2018 [32]	Ketamine for rapid reduction of suicidal thoughts in depression: Cognition and biomarkers in a midazolam-controlled trial	BDNF	RCT (*conference abstract- non-peer reviewed)	80; biomarkers: 53	MDD	KET (single infusion)	Peripheral blood (unspecified)	Baseline, 24 h post-infusion	≥50% reduction in SSI score from baseline 24 h after the infusion.	No significant changes.
Allen et al., 2014 [33]	Serum brain-derived neurotrophic factor and symptom improvements post-ketamine treatment in treatment-resistant depression	BDNF	Non-randomized interventional study (*conference abstract- non-peer reviewed)	17; 20 HC	TRD	KET 1–3 infusions	Serum	Baseline; 1 h, 24 h, 1 week post-infusion	No formal definition of clinical response; changes in MADRS score were analyzed.	No significant changes.
Mathew S.J et al., 2013 [34]	Ketamine and NMDA receptor modulation for treatment-resistant mood disorders	BDNF	RCT (*conference abstract- non-peer reviewed)	72	TRD	KET (single infusion)	Plasma	Baseline; 240 min post-infusion	No formal definition of clinical response.	Greater increase in BDNF levels after KET infusion in responders vs. non-responders, correlated with clinical improvement.
Zheng W et al., 2021 [35]	Association of plasma VEGF levels and the antidepressant effects of ketamine in patients with depression.	VEGF	Non-randomized interventional study	96	MDD	KET (6 infusions)	Plasma	Baseline, after 6th infusion	≥50% reduction in MADRS score from baseline on Day 13 (after six KET infusions).	No significant changes in VEGF levels following treatment; no differences between responders and non-responders; no correlation with clinical improvement.
Clarke et al., 2014 [36]	Biomarkers of the rapid clinical response to ketamine in treatment-resistant depression: Focus on kynurenine	KYNA	Non-randomized interventional study (*conference abstract- non-peer reviewed)	17; 20 HC	TRD	KET 1–3 infusions	Plasma	Baseline; 2 h; 24 h; 1 week post-infusions	No information on the definition of clinical response (conference abstract).	There were no differences in baseline TRP. KET did not alter KYN or TRP concentrations at any time point.
Yieh et al., 2017 [37]	Relationship of the kynurenine pathway to the improvement of depression in esketamine-treated subjects with major depressive disorder at imminent risk for suicide	KYNA	RCT (*conference abstract- non-peer-reviewed)	26; 22 placebo	MDD	ESK	Plasma	Baseline; 4 h; 1, 3, 25 days post-infusion	Not defined; changes in MADRS score were analyzed (4 h, Day 3, and Day 25).	Lower baseline TRP and 3-hydroxykynurenine (KYN3OH) levels were associated with a better response to ESK.
Petit et al., 2019 [38]	Kynurenine pathway as a target and biomarker of the antidepressant effect of ketamine	KYNA	Non-randomized interventional study (*conference abstract- non-peer reviewed)	15	TRD	KET (1–2 infusions per week)	Plasma	Before and after each infusion	≥50% reduction in MADRS score from baseline.	Higher baseline KYNA/QUIN ratio predicted a better antidepressant response to KET; reduced QUIN levels after infusion were associated with symptom improvement.
Spangemacher M et al., 2025 [39]	Biological and Clinical Stratification of Treatment-Resistant Depression: The Key Role of the Kynurenine Pathway in Ketamine Response	KYNA	Non-randomized interventional study (*conference abstract- non-peer reviewed)	32	TRD	KET 6 infusions	Peripheral blood (unspecified)	Baseline, 3, 18 days post-infusion	No information on the definition of clinical response (conference abstract).	Higher baseline KYNA levels correlated with greater clinical improvement.
A.P. Allen et al., 2018 [40]	Kynurenine pathway metabolism and the neurobiology of treatment-resistant depression: Comparison of multiple ketamine infusions and electroconvulsive therapy	KYNA, IL-6, IL-8, IL-10, INF	Observational study	37 (20 ECT, 17 KET); 20 HC	TRD	KET repeated infusions	Plasma	Baseline; 2 h, 24 h, 1 week post-infusions	≥50% reduction in MADRS score from baseline after completion of four KET infusions (2 weeks).	Symptom improvement correlated with a transient increase in KYNA levels; no significant changes in the other biomarkers.
Moaddel R et al., 2018 [41]	Plasma metabolomic profiling of a ketamine and placebo crossover trial of major depressive disorder and healthy control subjects.	KYNA, Arginine, Sphingomyelins	RCT	29, 25 HC	MDD	KET (single infusion)	Plasma	Baseline; 230 min; 1, 3 days post-infusion	≥50% reduction in MADRS score from baseline 230 min after the KET infusion.	KET induced early changes in the kynurenine and arginine pathways that distinguished responders from non-responders; no changes in sphingomyelins.
Yang JJ et al., 2015 [42]	Serum interleukin-6 is a predictive biomarker for ketamine’s antidepressant effect in treatment-resistant patients with major depression.	KYNA, IL-6, IL-1β, TNF-α	Non-randomized interventional study	16; 24 HC	MDD	KET (single infusion)	Serum	Baseline; 230 min; 1; 3 days post-infusion	Not reported; responders vs. non-responders analyzed, response criterion not specified.	Higher baseline IL-6 levels in responders; IL-6 and IL-1β decreased after KET only in responders; baseline IL-6 may predict KET response in TRD; no changes in KYNA metabolites or TNF-α.
Vande Voort J et al., 2023 [43]	Ketamine Associated Change in Anhedonia and Suicidal Ideation are Associated With Peripheral Immune Cell mTOR Expression and Kynurenine Levels and Treatment Resistant Depression	mTor	Non-randomized interventional study (*conference abstract- non-peer reviewed)	13; biomarkers:10	TRD	KET repeated infusions	PBMC	Baseline; after 1st infusion	No information on the definition of clinical response (conference abstract).	Increased mTOR expression correlated with reduced anhedonia.
Denk MC et al., 2011 [44]	Monitoring ketamine treatment response in a depressed patient via peripheral mammalian target of rapamycin activation.	mTOR	Case study	1	MDD	KET (single infusion)	PBMC	Baseline; several points not specified	Not applicable—case study; no formal definition of clinical response.	Increased p70S6K phosphorylation after KET paralleled rapid clinical improvement.
Yang C et al., 2013 [45]	Acute increases in plasma mammalian target of rapamycin, glycogen synthase kinase-3β, and eukaryotic elongation factor 2 phosphorylation after ketamine treatment in three depressed patients.	mTOR, GSK-3	Case series	3	MDD	KET (single infusion)	Plasma	Baseline; 10, 20, 30, 60, 120 min post-infusion	Not applicable—case series (3 patients); no formal definition of clinical response.	Progressive increases in p-mTOR and p-GSK-3β phosphorylation after KET; KET was associated with acute activation of the mTOR and GSK-3β pathways, paralleling clinical improvement.
Roy AV et al., 2021 [46]	Brain entropy and neurotrophic molecular markers accompanying clinical improvement after ketamine: Preliminary evidence in adolescents with treatment-resistant depression.	mTOR, GSK-3	Non-randomized interventional study	10	TRD	KET (6 infusions)	PBMC	Baseline, 2 h, after 6th infusion	≥50% reduction in CDRS-R score from baseline after six KET infusions (2 weeks).	Increased insulin-stimulated mTOR activation (p-mTOR/mTOR) after serial KET infusions occurred only in responders and correlated with greater reduction in depressive symptoms; increased GSK-3β phosphorylation also occurred only in responders and correlated with symptom improvement.
Veldman ER et al., 2021 [47]	P11 (S100A10) as a potential predictor of ketamine response in patients with SSRI-resistant depression	P11	RCT	30	MDD	KET (single infusion)	PBMC	Baseline, 1–2 days post-infusion	≥50% reduction in MADRS score from baseline 24 h after KET infusion.	Higher baseline p11 levels in cytotoxic T cells were significantly associated with greater reduction in depressive symptoms.
Chen MH et al., 2018 [48]	Rapid inflammation modulation and antidepressant efficacy of a low-dose ketamine infusion in treatment-resistant depression: A randomized, double-blind control study.	IL-6, CRP, TNF-α	RCT	71	TRD	KET (single infusion)	Serum	Baseline; 40 min; 240 min; day 3 and 7	No formal definition of clinical response; changes in MADRS score were analyzed.	Higher baseline IL-6 levels were associated with KET response; early post-infusion changes in IL-6 were not consistently associated with clinical improvement; early TNF-α reduction correlated with symptom improvement; no changes in CRP levels.
Zhou Y et al., 2021 [49]	Plasma inflammatory cytokines and treatment-resistant depression with comorbid pain: improvement by ketamine.	IL-6	RCT	66, 60 HC	TRD	KET (6 infusions)	Plasma	Baseline; day 13, 26	≥50% reduction in MADRS score from baseline on Day 13 (after six KET infusions).	IL-6 levels decreased after KET; higher baseline IL-6 levels were associated with better KET response.
Kruse JL et al., 2021 [50]	Depression treatment response to ketamine: sex-specific role of interleukin-8, but not other inflammatory markers.	IL-6, IL-8 IL-10, TNF-α, CRP	Non-randomized interventional study	46	TRD	KET (single infusion)	Plasma	Baseline; 24 h post-infusion	≥50% reduction in MADRS score from baseline 24 h after KET infusion	In women, lower baseline IL-8 levels and increased IL-8 after treatment were associated with clinical improvement; in men, increased IL-8 was associated with poorer response. No effects were observed for IL-6, IL-10, TNF-α, or CRP.
Wang Y et al., 2025 [51]	Inflammatory cytokines, cortisol, and anhedonia in patients with treatment-resistant depression after consecutive infusions of low-dose esketamine	IL-6, TNF-α, IL-4, cortisol	Non-randomized interventional study	60	TRD	ESK, 6x	Plasma	Baseline, after 6th infusion	≥50% reduction in MADRS score from baseline	After ESK, IL-6 and TNF-α levels decreased, whereas IL-4 levels increased; cortisol levels decreased. These changes were not significantly associated with clinical response (MADRS).
Matloka et al., 2021 [52]	P.0078 Inflammatory cytokine plasma levels correlation with MADRS score in patients with treatment-resistant major depression after dry-powder inhaled esketamine administration	Cytokines: IL-1β, IL-6, IL-17A, IL-17F, IL-22, TNF-α, IL-4, IL-10, IL-21, IL-23, IL-25, IL-31, IL-33, IFN-γ, sCD40L	RCT (*conference abstract- non-peer-reviewed)	88	TRD	ESK 4x	Plasma	Baseline; 4 h, 24 h, day 11	No formal definition of clinical response; changes in MADRS score were analyzed.	Higher baseline IL-10, IL-17A, IL-21, IL-31, and IFN-γ levels were observed in responders/remitters; antidepressant response correlated with cytokine changes 24 h after the last dose.
Sakurai et al., 2025 [53]	Acute and long-term effects of repeated ketamine infusions in treatment-resistant depression and associated metabolite changes	Cortisol, IL-6, IL-4, TNF-α	Non-randomized interventional study	60	TRD	KET, 6 infusions	Plasma	Baseline, 1 h after 6th infusion	No formal definition of clinical response; changes in MADRS score were analyzed.	After six KET infusions, cortisol, IL-6, and TNF-α levels decreased; none predicted clinical response.
Zhan Y et al., 2020 [54]	Alterations of multiple peripheral inflammatory cytokine levels after repeated ketamine infusions in major depressive disorder.	GM-CSF, fractalkine, ITAC, IFN- γ, IL-1 β, IL-2, IL-4, IL-5, IL-6, IL-7, IL-8, IL-10, IL- 12p70, IL-13, IL-17A, IL-23, MIP-1 β, MIP-3 α i TNF- α	Non-randomized interventional study	60	TRD	KET 6 infusions	Plasma	Baseline; 24 h and 14 days after 6th infusion	≥50% reduction in MADRS score from baseline on Day 13 (after six ketamine infusions).	Delayed reductions in pro- and anti-inflammatory cytokines were observed; IL-6 and IL-17A may serve as dynamic response biomarkers, while lower baseline ITAC levels predicted clinical improvement. TNF-α, IL-1β, and IL-6 levels decreased after KET infusions.
Cavalcanti-Ribeiro et al., 2026 [55]	Inflammatory response to repeated subcutaneous esketamine in treatment-resistant depression: The role of baseline C-reactive protein.	CRP	Non-randomized interventional study	22	TRD	ESK 7x (subcutaneously)	Serum	Baseline; weeks 4, 8	No formal definition of clinical response; changes in MADRS score were analyzed.	In patients with elevated baseline CRP, CRP levels decreased significantly after treatment; no significant changes were observed in those with low baseline CRP. Changes in CRP did not predict MADRS improvement.
Kadriu B et al., 2018 [56]	Acute ketamine administration corrects abnormal inflammatory bone markers in major depressive disorder.	RANKL	RCT	28, 16 HC	MDD	KET (single infusion)	Plasma	Baseline, 230 min, day 1, 3	≥50% reduction in MADRS score at any post-infusion time point (230 min, Day 1, or Day 3).	KET normalized the dysregulated OPG/RANKL and OPN profile in patients with MDD, suggesting beneficial effects on inflammation and bone remodeling, with no predictive value for clinical response.
Naughton et al., 2014 [57]	The cortisol awakening response in treatment-resistant depression is not a biomarker of the clinical response to ketamine	Cortisol	RCT (*conference abstract- non-peer-reviewed)	17; 20 HC	TRD	KET 1–3 infusions	Saliva	Baseline; 24 h post-infusions	No formal definition of clinical response (conference abstract).	No significant changes.
Georgiou P et al., 2025 [58]	Associations between hypothalamic–pituitary–adrenal (HPA) axis hormone levels, major depression features and antidepressant effects of ketamine.	HPA axis hormones (CRF, ACTH, cortisol)	RCT	42, 23 HC	TRD	KET (single infusion)	Plasma	Baseline (pre-infusion)	No formal definition of clinical response; changes in MADRS score were analyzed.	Baseline CRF, ACTH, and cortisol levels were not associated with treatment response.
Greene B.D et al., 2026 [59]	Thyroid-stimulating hormone is associated with differential antidepressant and anti-anhedonic response to ketamine in bipolar depression but not major depressive disorder	TSH	RCT	39	MDD	KET (single infusion)	Serum	Baseline, 230 min; day 1, 2, 7	No formal definition of clinical response; changes in MADRS score were analyzed.	Baseline TSH levels were not associated with KET response in MDD; in bipolar depression, higher baseline TSH levels predicted greater reductions in depressive symptoms and anhedonia after KET treatment.
Tye S et al., 2023 [60]	Critical Role of Insulin as an Immunomodulatory Target in Treatment Resistant Depression	Insulin signaling/glucose uptake	Non-randomized interventional study (conference abstract- non-peer reviewed)	68	TRD	KET	Plasma	Not specified	No formal definition of clinical response; changes in MADRS score were analyzed.	Altered insulin signaling in PBMCs was associated with KET response; modulation of the insulin signaling pathway may enhance treatment efficacy.
Moaddel R et al., 2015 [61]	D-serine plasma concentration is a potential biomarker of (R,S)-ketamine antidepressant response in subjects with treatment-resistant depression.	D-Serine	Non-randomized interventional study	21	MDD	KET (single infusion)	PBMC	Baseline; 230 min; 21 days	≥50% reduction in MADRS score from baseline at 230 min post-infusion.	Lower baseline plasma D-serine levels were observed in responders to KET. Baseline plasma D-serine levels were significantly associated with the percentage change in MADRS score.
Zhou N et al., 2025 [62]	Proteomic patterns associated with ketamine response in major depressive disorders	Proteomic markers	Non-randomized interventional study	30	MDD	KET 6 infusions	Plasma	Baseline, after 6th infusion	≥50% reduction in total HAMD score from baseline after 2 weeks of treatment (following six KET infusions).	KET response in MDD was associated with regulation of plasma proteins involved in immune function. Three pretreatment proteins (PI16, NEO1, and TNXB) showed potential as predictive biomarkers of treatment response.
You et al., 2025 [63]	The Restoration of Energy Pathways Indicates the Efficacy of Ketamine Treatment in Depression: A Metabolomic Analysis.	ADP, ATP, pyruvate; FT3	Non-randomized interventional study	40	MDD	KET 6 infusions	Plasma	Baseline, day 13	≥50% reduction in total MADRS score from baseline at the end of treatment (Day 13, after six KET infusions).	KET modulated energy metabolism (ATP/ADP), and the magnitude of ADP changes correlated with clinical improvement. Lower baseline FT3 levels may serve as a predictive biomarker of KET response.
Singh B et al., 2026 [64]	Metabolomic signatures of intravenous racemic ketamine in treatment-resistant major depressive disorder: the Bio-K/NNDC multicenter trial	Metabolomic signature (acylcarnitines, AA, GABA, serotonin, NO markers)	Non-randomized interventional study (conference abstract—non-peer-reviewed)	69	MDD	KET, 3 infusions	Plasma	Baseline; after 1 and 3 infusions	No formal definition of clinical response	KET induced broad metabolic changes; greater increases in GABA synthesis in remitters; individual metabolites showed limited predictive value for treatment response.
Quintanilla et al., 2023 [65]	κ-Opioid Receptor Plasma Levels Are Associated with Sex and Diagnosis of Major Depressive Disorder but Not Response to Ketamine	Levels of κ-OR opioid receptors and dynorphins	RCT	39, 25 HC	MDD	KET	Plasma	Baseline; 230 min, day 1 and 3 post-infusion	No formal definition of response; change in MADRS score analyzed.	Plasma KOR and dynorphin levels differentiated MDD patients from healthy controls but did not predict KET response or change significantly after treatment.

Abbreviations: KET: ketamine; ESK: esketamine; MDD: Major Depressive Disorder; TRD: Treatment-resistant Depression; HC: healthy control; PBMC: peripheral blood mononuclear cells., *: Conference abstract (non-peer-reviewed).

**Table 2:** Summary of current evidence on peripheral biomarker categories associated with response to ketamine and esketamine in major depressive disorder.

Biomarker Category	Principal Biomarkers	Studies (Ketamine/Esketamine, *n*)	Main Findings	Overall Strength of Evidence	Key Research Gaps
Neuroplasticity	BDNF, VEGF, FGF-2, S100B	16/1	BDNF is the most extensively investigated biomarker; however, baseline concentrations and treatment-induced changes remain inconsistent across studies.	★★★☆☆	Standardization of sampling procedures and validation in esketamine-treated patients.
Inflammation	IL-6, TNF-α, IL-10, IL-8, CRP, IFN-γ and other cytokines	9/3	IL-6 appears to be the most promising inflammatory biomarker, although findings remain heterogeneous and difficult to reproduce.	★★★☆☆	Large multicenter studies and harmonized assessment of inflammatory markers.
Kynurenine pathway	TRP, KYN, KYNA, QUIN, 3-HK	6/1	Several studies suggest predictive potential, particularly for KYNA-related measures, but evidence remains limited.	★★★★☆	Independent validation and standardized metabolomic protocols.
Neuroendocrine markers	Cortisol, TSH, fT3	5/1	Available studies provide inconsistent evidence regarding endocrine predictors of treatment response.	★★☆☆☆	Larger cohorts and studies involving esketamine-treated patients.
Intracellular signaling	mTOR, GSK-3, p11	5/0	Preliminary findings support the involvement of intracellular signaling pathways; however, evidence is based mainly on exploratory studies.	★★☆☆☆	Replication in adequately powered clinical studies.
Metabolomic/proteomic markers	Arginine, sphingomyelins, proteomic signatures	4/0	Current evidence is exploratory and insufficient to identify clinically useful biomarkers.	★☆☆☆☆	Comprehensive multi-omics studies integrating biological and clinical data.

Abbreviations: TRP, tryptophan; KYN, kynurenine; KYNA, kynurenic acid; QUIN, quinolinic acid; 3-HK, 3-hydroxykynurenine. Overall strength of evidence (★☆☆☆☆–★★★★★) represents the authors’ qualitative assessment based on the number of available studies, consistency of findings, study design (including the presence of randomized controlled trials), replication across independent cohorts, sample size, and methodological robustness. This rating is intended solely as a descriptive summary of the current literature and does not represent a formal assessment of certainty or quality of evidence (e.g., GRADE).

## Data Availability

No new data were created or analyzed in this study. Data sharing is not applicable to this article.

## Supplementary Materials

The following supporting information can be downloaded at: https://www.mdpi.com/article/10.3390/jpm16100488/s1, Table S1. Detailed summary of peripheral biomarkers investigated as predictors or correlates of response to ketamine and esketamine in major depressive disorder; PRISMA-ScR Checklist: Preferred Reporting Items for Systematic Reviews and Meta-Analyses Extension for Scoping Reviews (PRISMA-ScR) Checklist.
