# Supplementary material for: Peripheral Biomarkers of Response to Ketamine and Esketamine Treatment in Unipolar Depressive Disorder—A Scoping Review
Source: J Pers Med. 2026 Sep 23;16(10):488. doi: 10.3390/jpm16100488 (PMC13706079; doi:10.3390/jpm16100488)
Supplement: Supplementary file 1 [file jpm-16-00488-s001.zip › jpm-4506100-supplementary-Table S1.pdf]

Table S1. Detailed summary of peripheral biomarkers investigated as predictors or correlates of response to ketamine and esketamine in major depressive disorder

Biomarker	Studies	RCTs	Positive studies	Ketamine	Esketamine	Main role	Overall evidence	Main limitations
**BDNF**	15	6	6	14	1	Predictive & dynamic	★★★☆☆	Highly heterogeneous findings
**IL-6**	10	3	4	8	2	Mainly predictive	★★★☆☆	Poor reproducibility
**TNF-α**	9	2	2	8	1	Dynamic	★★☆☆☆	Mostly negative studies
**KYNA**	7	2	5	6	1	Predictive	★★★★☆	Limited replication
**IL-10**	5	1	1	5	0	Dynamic	★★☆☆☆	Small cohorts
**IL-8**	4	0	1	3	1	Dynamic	★★☆☆☆	Few studies
**CRP**	3	1	1	3	0	Predictive	★☆☆☆☆	Insufficient evidence
**Cortisol**	3	2	0	3	0	Predictive	★☆☆☆☆	Negative findings
**mTOR**	4	0	4	4	0	Dynamic	★★☆☆☆	Very small studies
**GSK-3**	2	0	2	2	0	Dynamic	★☆☆☆☆	Only two studies
**Other biomarkers**	1 each	–	–	–	–	Exploratory	★☆☆☆☆	Single-study evidence

Overall evidence represents the authors' qualitative assessment based on the number of available studies, proportion of significant findings, study design (including RCTs), consistency of results across studies, and sample size. This rating is intended as a descriptive summary and does not represent a formal GRADE assessment.
